# Prevalence of latent tuberculosis infection and associated risk factors in an urban African setting

**DOI:** 10.1186/s12879-015-0904-1

**Published:** 2015-03-29

**Authors:** Florence N Kizza, Justin List, Allan K Nkwata, Alphonse Okwera, Amara E Ezeamama, Christopher C Whalen, Juliet N Sekandi

**Affiliations:** Department of Epidemiology and Biostatistics, College of Public Health, University of Georgia, Athens, GA USA; Department of Internal Medicine University of Michigan, Ann Arbor, MI USA; Makerere University School of Public Health, Kampala, Uganda; National TB Treatment Center, School of Medicine, Makerere University, Mulago, Uganda

**Keywords:** Latent tuberculosis infection, Tuberculin skin testing, TB control

## Abstract

**Background:**

Nearly one third of the world is infected with latent tuberculosis infection (LTBI) and a vast pool of individuals with LTBI persists in developing countries, posing a major barrier to global TB control. The aim of the present study was to determine the prevalence of LTBI and the associated risk factors among adults in Kampala, Uganda.

**Methods:**

We performed a secondary analysis from a door-to-door cross-sectional survey of chronic cough conducted from January 2008 to June 2009. Urban residents of Rubaga community in Kampala aged 15 years and older who had received Tuberculin skin testing (TST) were included in the analysis. The primary outcome was LTBI defined as a TST with induration 10 mm or greater. Multivariable logistic regression analyses were used to assess the risk factors associated with LTBI.

**Results:**

A total of 290 participants were tested with TST, 283 had their tests read and 7 didn’t have the TST read because of failure to trace them within 48–72 hours. Of the participants with TST results, 68% were female, 75% were 15–34 years, 83% had attained at least 13 years of education, 12% were smokers, 50% were currently married, 57% left home for school or employment, 21% were HIV positive and 65% reported chronic cough of 2 weeks or longer. The overall prevalence of LTBI was 49% [95% CI 44–55] with some age-and sex-specific differences. On multivariable analysis, leaving home for school or employment, aOR = 1.72; [95%CI: 1.05, 2.81] and age 25–34, aOR = 1.94; [95%CI: 1.12, 3.38]; 35 years and older, aOR = 3.12; [95%CI: 1.65, 5.88] were significant risk factors of LTBI.

**Conclusion:**

The prevalence of LTBI was high in this urban African setting. Leaving home for school or employment and older age were factors significantly associated with LTBI in this setting. This suggests a potential role of expansion of one’s social network outside the home and cumulative risk of exposure to TB with age in the acquisition of LTBI. Our results provide support for LTBI screening and preventive treatment programs of these sub-groups in order to enhance TB control.

## Background

Tuberculosis (TB) ranks the second leading cause of mortality among infectious diseases worldwide after the human immunodeficiency virus (HIV) [1]. In 2012, the World Health Organization (WHO) estimated 8.6 million new TB cases and 1.3 million TB deaths. Over 95% of the TB cases and 98% of deaths occurred in low and middle income countries. According to the WHO, Uganda ranks the 16^th^ among the 22 high burden countries for TB disease [2].

Latent tuberculosis infection (LTBI) refers to a condition in which viable *Mycobacterium tuberculosis* (MTB) bacilli are present in an individual without manifestation of clinical symptoms and signs of active disease [3]. Nearly one third of the world’s population is infected with LTBI [4]. People infected with LTBI are not infectious, but they are at risk of developing active disease and becoming infectious. Studies show that 5–20% of those infected will progress to active TB at some point in their lifetime, with the majority developing TB disease within 2–5 years of the initial infection [5].

LTBI is highly prevalent in developing countries and poses a major barrier to global TB control [6]. Identification of such people will increase case detection rates and may dictate new treatment policies to control tuberculosis [7]. Various studies have investigated the prevalence of LTBI in healthcare workers, prisons and gold miners which are high risk populations [8-14] but few community-based studies [15,16] have considered urban populations. To determine the prevalence of LTBI and the associated risk factors in an urban African setting we conducted a secondary analysis of a house-to-house cross-sectional survey. Information from this study will be of utility to the national TB control programs for increasing LTBI screening in the general population and enhancing TB control through targeted allocation of resources to highest risk population segments.

## Methods

### Study setting and population

This study was a secondary analysis of a house-to-house cross-sectional survey of chronic cough that was conducted in Rubaga, an urban setting in Uganda, from January 2008 to June 2009. Rubaga is located in Kampala, the capital district of Uganda and is one of the five administrative divisions with a population of about 400,000 people, 50% of whom are adults 15 years and older. It is subdivided into 13 parishes and 127 villages (zones) with an estimated total of 75,485 households [17]. The division is served by two private hospitals, two public health centres and several private clinics which offer TB, HIV and general health services. Residents were eligible to participate in the survey if they were aged 15 years and older and were members of households visited during the survey period. Individuals were excluded from the analysis if they did not receive Tuberculin skin testing (TST) using the Mantoux method or were missing test results if tested.

### Ethical approval

The primary study was approved by the Higher Degrees Research Ethics Committee (HDREC) at Makerere University School of Public Health and the Uganda National Council for Science and Technology. All eligible participants provided informed written informed consent or assent from parents for those who were 15–17 years. Participants who were less than 18 years but living on their own or heading households were eligible for enrolment as emancipated minors. A detailed description of the primary study methods of the survey are available elsewhere [18].

### Sampling of study population

In the underlying study, 5,102 participants were selected using a multi-stage sampling approach. A simple random sample was used to select five of the 13 parishes using a computer-based random number generator and sampling frame from the Uganda Bureau of Statistics. Weighted proportions-to-village population sizes were calculated to estimate the number of participants to be recruited from each village. This was done to account for the variability in crowding. We identified the first house from a defined central point such as road or drainage junctions in each village and enrolled a convenience sample of persons at home. During the period we also randomly sampled 100 controls, defined as individuals who did not report chronic cough using computer-generated random numbers.

### Laboratory methods

Rapid HIV testing was performed using the serial algorithm based on the Uganda Ministry of Health recommendation. This sequential test includes first the Determine HIV-1/2 assay (Abbott Laboratories, Illinois, United States of America) for screening followed by the HIV-1/2 STAT-PAK Dipstick assay (Chembio Diagnostic System Inc, New York, USA) for confirmatory testing. An HIV-negative result with the Determine assay was reported as negative. An HIV-positive with the Determine assay was confirmed positive on the STAT-PAK assay. If the Determine and STAT-PAK assays were discordant, a third test was performed, Uni-Gold test (Trinity Biotech, Wicklow, Ireland) was performed; the results were reported as positive if the Uni-Gold test was positive and negative if both STAT-PAK assay and Uni-Gold results were negative. If results were indeterminate on the tie-breaker or there was discordance in the interpretation of results by the home visitors, a confirmatory PCR test would be done at a local reference laboratory.

Tuberculin skin testing (TST) was performed on chronic coughers and non-chronic cougher controls by placing 0.1 ml of 5 tuberculin units of purified protein derivative (Tubersol; Connaught Laboratories, Limited, Toronto, Canada) on the left forearm using the Mantoux method. Between 48 and 72 hours, the diameter of palpable skin induration was recorded (in millimeters) independently by two home visitors using digital calipers. A tuberculin skin test was be considered positive if it was 5 mm or greater for the HIV-infected and 10 mm or greater for the HIV sero-negative participants.

### Statistical analysis

We performed secondary data analysis to determine the prevalence of LTBI and the risk factors associated with LTBI in Rubaga district of Kampala among the 283 participants enrolled as part of the house-to-house cross-sectional survey. The primary outcome for this study was presence of latent TB infection (LTBI), defined as a tuberculin skin test (TST) induration ≥10 mm [19]. The potential risk factors considered were; age [15–24, 25–34 and ≥35 years], leaving home for school or employment, sex, marital status, smoking status, HIV status and chronic cough defined as self-reported cough ≥ 2 weeks at the time of the survey.

The overall prevalence of LTBI was estimated by dividing the number of participants with TST ≥10 mm by the total number of study participants who had undergone the TST test. Frequencies and percentages were used to summarize descriptive characteristics for the study population. For categorical variables, the chi-square test was used to test the difference in proportions of LTBI. Using bivariate analysis crude odds ratios and corresponding 95% confidence intervals (95% CI) were determined. A *p-value* <0.2 for the unadjusted association between a risk factor and LTBI was considered suggestive of potential significance. We included all variables with *p-value* <0.2 and other potential confounders based on previous studies [12,15] in multivariable models. Multiple logistic regression analysis was performed to assess simultaneously the association between multiple risk factors and LTBI. From this model, adjusted ORs and 95% CI were calculated. We looked at covariates using a stepwise regression model, and noted variables that held a significant association with LTBI at <0.05. All the analyses were done using SAS version 9.1.3.

## Results

### Baseline study population

Overall, there were 290 participants that were tested with TST. Of those 283 had their tests read and 7 participants did not have the TST read because they were untraced within 48–72 hours window of TST reading of whom 4 were male and 3 were female. Of 283 individuals who were tested with TST, 68% were female, 75% were 15–34 years, 83% had attained at least 13 years of education, 12% were smokers, 50% were currently married, 57% left home for school or employment and 21% were HIV positive and 65% reported chronic cough ≥ 2 weeks while the 35% were no chronic cough controls (Table 1).

**Table 1:** **Characteristics of the study population with a TST reading (N = 283)**

**Characteristic**	**Frequency (%)**
Sex	
Male	91(32.2)
Female	192(67.8)
Age group(years)	
15-24	111(39.2)
25-34	103 (36.4)
35+	69(24.4)
Education (Years)	
No education	28(9.9)
1-7	137(48.4)
8-13	100(35.3)
>13	18(6.4)
Marital status	
Never married	73(25.8)
Currently married	141(49.8)
Previously married	69(24.4)
Religion	
Catholic	101(35.7)
Protestant	87(30.7)
Muslim	64(22.6)
Other*	31(11.0)
Smoking Status	
Never smoker	228(80.6)
Previous smoker	21(7.4)
Current smoker	34(12.0)
HIV status	
Negative	221(78.9)
Positive	59(21.1)
Missing	3
Leave home for school or employment	
Yes	162(57.2)
No	121(42.8)
Chronic Cough (> = 2 weeks)	
Yes	183(64.7)
No	100(35.3)

Other* = Pentecostal, Seventh Day Adventists, Religion not specified.

### Prevalence of LTBI

**Figure 1:**
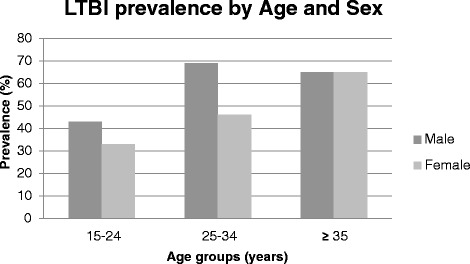
**Age- and sex-specific LTBI prevalence.**

In this study the overall prevalence of LTBI was 49% [95% CI, 44–55]. Overall, LTBI prevalence also increased with age, from 36% in the 15–24 age group to 65% in the 35 and older age group. Stratifying by sex, the prevalence of LTBI was higher in males as compared to females in the 15–24 and 25–34 year age groups (43% vs 33% & 69% vs 46%). However, there was no sex-specific difference in LTBI among the 35 and older age group (Figure 1). The prevalence of LTBI was also higher in those who were employed or students (56%) compared to those who were not (40%) and for those who had chronic cough (54%) compared to those who did not (42%). Similarly, the prevalence was higher in current (65%) or previous smokers (52%) compared to never smokers (47%) and currently (54%) or previously married (53%) compared to never married (36%) (not inTable 2). As reported in the primary study, there were 39 active TB cases of whom 28 (20%) were TST positive (> = 10 mm induration). Also, 13 eligible participants had previous history of TB, 12 had TST readings, of whom 5 (42%) were TST positive. There were 14 people in current TB treatment but none of them received TST because it was irrelevant (not included in Table 2).

**Table 2:** **Logistic regression analysis of risk factors associated with LTBI**

**Risk factor**	**TST ≥ 10 mm**	**TST < 10 mm**		
	**N (%)**	**N (%)**	**Unadjusted OR (95% CI)**	**Adjusted OR (95%CI)**
	**140(49)**	**143(51%)**		
**Sex**				
Male	54(39)	37(26)	**1.80(1.08,2.98)**	
Female	86(61)	106(74)	1.00	
**Age group (years)**				
15-24	40(29)	71(50)	1.00	1.00
25-34	55(39)	48(33)	**2.03(1.78,3.52)**	**1.94(1.12,3.38)**
35+	45(32)	24(17)	**3.33(1.77,6.24)**	**3.12(1.65,5.88)**
**Education (years)**				
No education	13(9)	15(10)	1.00	
1-7	73(52)	64(45)	1.32(0.58,2.97)	
8-13	44(31)	56(39)	0.91(0.39,2.10)	
>13	10(7)	8(6)	1.44(0.44,4.74)	
**Marital Status**				
Never married	26(19)	47(32.9)	1.00	
Currently married	76(54)	65(45.4)	**2.11(1.18,3.78)**	
Previously married	38(27)	31(29.7)	**2.22(1.13,4.35)**	
**Religion**				
Catholic	44(31)	57(40)	0.82(0.38,1.85)	
Protestant	47(34)	40(28)	1.25(0.55,2.85)	
Muslim	34(24)	30(21)	1.21(0.51,2.85)	
Other*	15(11)	16(11)	1.00	
**Smoking status**				
Never smoker	107(76)	121(85)	1.00	
Previous smoker	11(8)	10(7)	1.24(0.51,3.04)	
Current smoker	22(16)	12(8)	2.07(0.98,4.39)	
**HIV status**				
Negative	110(80)	111(78)		
Positive	28(20)	31(22)	0.91(0.52,1.62)	
**Missing**	3	1		
**History of TB disease**				
Yes	7(4)	5(4)	0.72(0.22,2.32)	
No	136(96)	135(96)	1.00	
**Leave home for school or employment**				
Yes	91(65)	71(50)	**1.88(1.17,3.04)**	**1.72(1.05,2.81)**
No	49(35)	72(50)	1.00	1.00
**Chronic cough(> = 2 weeks)**				
Yes	98(70)	85(59)	1.59(0.97,2.60)	
No	42(30)	58(41)	1.00	

TST: Tuberculin Skin Test OR = Odds Ratio CI = Confidence Interval.

Bold numbers are statistically significant findings.

### Factors associated with LTBI

The bivariate analysis showed that the risk of LTBI was higher among those aged 25–34 years (OR = 2.03, 95%CI: 1.78, 3.52) and ≥35 years (OR = 3.33, 95% CI: 1.77, 6.24) compared to those aged 15–24 years. Similarly, the risk was higher in individuals currently married (OR = 2.13, 95% CI: 1.17, 3.89) and previously married (OR = 1.99, 95% CI: 1.02, 3.87) as compared to those who were never married. Additionally, the risk of LTBI was higher among those who left home for school or employment (OR = 1.88, CI: 1.17, 3.04) compared those who were not and higher in males (OR = 1.80, 95% CI: 1.08, 2.98) as compared to females (Table 2).

In a series of multivariable analyses, we initially considered HIV status, smoking, marital status, chronic cough and sex as potential confounders based on previous studies and biologic plausibility but none of them were included in the final model because they were not found to be confounders in our analysis. We looked at covariates using a stepwise regression model, and noted variables that had a significant association with LTBI at a p-value less than 0.05. Therefore, we selected the final model with two covariates; Age and individuals employed or students (indicating leaving home daily for work or school) based on a measure of model goodness of fit, the Akaike Information Criterion (AIC). Leaving home for school or employment was associated with LTBI (OR = 1.72, 95% CI: 1.05, 2.81) and age 25–34 years old (OR = 1.94, 95% CI: 1.12, 3.38) and ≥35 years (OR = 3.12, 95% CI: 1.65, 5.88) (Table 2).

## Discussion

In this study of adult participants from an African urban setting, nearly half of the population was infected with mycobacterium tuberculosis. Leaving home for school or employment and age were significantly associated with LTBI. The prevalence of LTBI found in this study was consistent with other studies done in Peru in urban and peri-urban populations that found the prevalence of LTBI to be 50-52% [16,20]. In contrast, the prevalence of LTBI was higher than from community based studies conducted in other African settings that found the prevalence of LTBI to be ranging from 31% in Ethiopia to 40% in Zambia [15,21]. Additionally, the prevalence was lower than that reported from an adult population-based study in South Africa [22].

The study showed that leaving home for school or employment was associated with latent TB infection, suggesting the possibility of exposure outside of home. It is plausible that as people frequently move outside of their homes, there is an expansion of the social network with which they mix hence the possibility of greater exposure to TB compared to individuals who have limited exposure within their home and immediate neighborhood. There is mounting evidence to support the role of social networks in the transmission of tuberculosis [23-26] and other infectious diseases such as HIV and other sexually transmitted infections [27]. Our study findings allude to a possibility of mixing with social networks outside the home at places of employment or school. Evidence from different studies shows conflicting results on where LTBI appears to be transmitted most. For instance, studies done in Uganda, Thailand, India and China have reported more transmission at home due to household contact exposure [28-34]. However, other studies have shown that more transmission happens outside home especially in work places like healthcare settings due to nosocomial transmission [9,10,13]. Others demonstrate higher transmission among students in the healthcare settings, prisoners and gold miners [11,14,35] which are higher risk populations. To the best of our knowledge, our study is among the first to show that the risk of LTBI is higher among those who are employed and students in the general population.

The results of the multivariable analysis showed a dose–response relationship between age and LTBI, with a 2- to 3-fold increase in risk for LTBI from younger to older age. This may denote a cumulative lifetime risk of infection. This finding is consistent with studies done in Kampala, Uganda, South Africa and Peru that found that the prevalence of LTBI among community members increased with age [20,22,28] . The higher risk of LTBI in the older age group may be due to waning effect of BCG vaccination with increasing age and may also reflect the increase in cumulative exposure to TB with age.

In most high burden settings, less emphasis is placed on screening and treatment of LTBI in general populations and preventive therapy remains underutilized in the HIV-infected population as a method of TB control [36,37]. The main challenge associated with implementation of LTBI screening and treatment relate to ensuring proper exclusion of active TB disease and balanced against the limited resources available for treating active TB cases [38,39]. The high prevalence of LTBI in our study highlights the need to strengthen TB control efforts that focus on preventive therapy to curtail reactivation to active TB disease among populations. In light of prevailing resource constraints health programs, it is plausible to target LTBI screening and treatment to populations with the highest risk of reactivation or progression to TB disease such the HIV-infected, children under-five years and recently exposed individuals. Our study suggests that people who are employed or those who are students had significantly greater odds of LTBI as shown by a positive tuberculin skin test, than those who stayed at home, moreover the overall proportion of HIV infection was as high as 20%. Previous studies have shown that employment categories at highest risk include healthcare workers and gold miners [8,9,14,40]; participants in our study worked in diverse setting including mainly trade and outdoor markets but none were in the known high-risk categories. Our findings and those from previous studies suggest a potential need for periodic LTBI screening coupled with preventive treatment programs at workplaces but the cost-effectiveness of such an intervention should be evaluated.

### Strengths and limitations

The strength of this study is that it provides a relative precise snap shot of the prevalence of LTBI in an urban African setting as it was a population-based study conducted in an urban community. Another unique feature of this study is that it demonstrates the risk of LTBI being higher in those who leave home for school or employment from the general population. On the other hand, this was a secondary analysis of cross-sectional study that was initially designed to answer a different primary research question hence subject to some limitations. By design, this analysis we could only answer questions based on the available data. For example, information on history of recent close contact with TB was not collected even though it’s well known predictor of LTBI. Also, the participants’ identifications were not specifically linked to the households from which they were sampled making it impossible to account for the potential household clustering effect on the prevalence of LTBI. We expect that if the clustering effect was indeed present, it could have led to a slight overestimation of the measures of association between covariates and the primary outcome (LTBI). Additionally, information on chronic cough was self-reported therefore subject to recall bias; this may have resulted in misclassification and exclusion of some participants who could have been eligible for TST therefore resulting in the underestimation of the prevalence LTBI. Furthermore, the limited sensitivity of tuberculin skin test may have under-estimation of the true prevalence of LTBI however; we believe that this is a valuable addition to the few existing population-based estimates of prevalence of LTBI in this urban setting.

## Conclusion

The prevalence of LTBI was high in this urban African setting. Leaving home for school or employment and older age were associated with LTBI suggesting a potential underlying risk related to expansion of a person’s social network outside the home where exposure to TB infection is likely to occur and cumulative risk with age. Our results provide support for LTBI screening and preventive treatment programs of these sub-groups in order to enhance TB control.

## Abbreviations

LTBI: Latent tuberculosis infection
MTB: *Mycobacterium tuberculosis*
HIV: Human immunodeficiency virus
WHO: World Health Organization
DOTS: Directly observed therapy short-course.
